# Lungs and Air

**Published:** 1869-11

**Authors:** 


### LUNGS AND AIR.

SCIENTIFIC men have computed, that if every air-cell in the lungs were opened and spread out on a wall, over four hundred square feet of surface would be covered, which is many times larger than the skin would cover.

If the open hand be put in water, and then be suddenly placed on any part of the body, the shock of the coldness is such, that it produces a very disagreeable sensation all through the system.

But if, immediately on coming out of a warm apartment, a bucket of water were dashed over the skin, the present shock, and the subsequent ill effects, might well nigh prove fatal in many cases, especially if additional bucketfuls were thrown on, at intervals of a quarter of a minute.

The lungs are not as sensitive as the skin, but they are very susceptible of impressions from cold. A man in good health breathes sixteen times in a minute, and sixteen times in a minute does the air taken in come in direct contact with the inner surface of the lungs. If then we go into a very cold, raw air, from a warm room, engaging in conversation, every breath of cold air dashes right through the open mouth, directly in upon the surface of the heated lungs—a difference of sixty degrees, if there be ice or snow about, and greater still if the wind is blowing. The rapidity with which a chill passes over the body, on passing from a warm room to a piercing atmosphere, is positive proof of the great effect produced. Hence, it is not at all wonderful, that persons, in robust health even, "catch their death of cold" by such exposures, in the shape of pleurisies, lung fever, pneumonia, bad colds, and terrible chills.

In almost all cases, these ill results may be averted, or greatly modified, if the person will take two precautions. First, close the mouth before passing out, thus compelling the air to pass, by the circuit of the nose, through the head, and thus be measurably warmed before it reaches the lungs. Second, walk rapidly, so as to throw the blood to the surface, but soon abate the gait, as we cannot get air enough through the nose, only for a few minutes; or hold a handkerchief over the nose and open mouth, in the shape of a loose ball, so as to allow the cold and hot air to commingle in its folds. This may suggest a reason for allowing the beard to grow around the lower part of the face, as, in the first place, it impedes the ingress of cold air and the egress of the warm air from the lungs, and thus commingled, the cold air is modified. A beard is not given to women, because every woman ought to have a home and a husband; and it is her business to stay there, and care for, and make things tidy, while he is out, and about, spending his energies in providing the means for his wife's comfort and happiness.

By some means, nature is sometimes thwarted, or perverted, and monstrosities result, anomalies, or as doctors call them, idiosyncrasies—the consequence being a beardless man, or a bearded woman. Whether all women's rights women have pretty much of a beard or not, we do not know; some of them we have seen on the rostrum—the leading spirits—and they made a pretty good show of masculinity on the upper lip. Certain it is, that such appearances indicate a masculine nature; and the want of a beard, an effeminacy of temperament, when observed in men. Hence, we infer that the proper place for a woman—the place where she appears most glorious—is, with her children around her, at the domestic board, at the bedside of suffering, or in her "Father's House," yielding Him the worship of her inmost heart. But if instead of making herself thus sweetly glorious, she fancies the forum and the hustings, and ruthlessly exposes herself to the ribald gaze of the vulgar crowd, it is but a step to that deeper depth of contempt or degradation to which, sooner or later, she seldom fails to arrive.

